# Prevalence of Renal Osteodystrophy in African Hemodialysis Patients

**DOI:** 10.5812/numonthly.9398

**Published:** 2013-06-01

**Authors:** Abdelaali Bahad, Driss El Kabbaj, Mohammed Benyahia

**Affiliations:** 1 Department of Nephrology, Dialysis and Kidney Transplantation, Military Teaching Hospital Mohammed V, Rabat, Morocco

**Keywords:** Renal Osteodystrophy, Renal Dialysis, Africa

*Dear Editor,*

Disturbances in mineral and bone metabolism are common in patients with chronic kidney disease and it increases with progression of renal insufficiency. Renal osteodystrophy (ROD) is a general term encompassing both high and low bone turnover forms of bone disease. The overall incidence of ROD in patients with advanced renal failure and those treated with maintenance hemodialysis (HD) is 90 to 100% (1). The nature and type of ROD varies from one patient to another and several factors may account for this variation (2). The two most commonly encountered types of ROD are: high turnover hyperparathyroid and low turnover ABD. Bone biopsy is the gold standard; however, serum levels of PTH are considered an adequate screening tool to separate these two diseases and it is associated to cardiovascular morbidity and mortality (2).

In Africa, information on ROD is sparse and very different. Buargub and et al. find hyperparathyroidism in 29 of 103 Libyan patients (28.1%) while 28 patients (27.1%) had laboratory evidence of adynamic bone disease (3). Sek and et al. report, in Senegal, that 57 of 118 cases (48.3%) had secondary hyperparathyroidism (SHPT) and 21 with adynamic bone disease (17.8%) (4).

The limitations of these two studies were: bone biopsy was not available and authors depended completely on plasma PTH levels in diagnosis of renal bone disease. The second limitation is that the precision of the type of biological method of PTH dosage. They were not able to measure serum levels of 25-OH vitamin D and aluminum to exclude the possibility of osteomalacia due to vitamin D deficiency or aluminum toxicity. Finally the authors used the national kidney disease Foundation-kidney disease outcomes quality initiative (K/DOQI) guidelines published in 2003 to define ROD that major prevalence of hyperparathyroidism than the Kidney disease improvement global outcomes guidelines (KDIGO).

A Nigerian report (5) shows that there were hypocalcaemia and hyper-phosphataemia in 80% and 60% of the patients respectively. Alkaline phosphatase was elevated in 44% of the patients while 11.8% had hyperparathyroidism. Level of 25 (OH) 2 Vitamin D3 was low in 83.3% of the patients. 

Finally it is very important to say that prevalence and type of ROD depend on medical staff attitude. In the past two decades, the prevalence of high turnover ROD has decreased while low bone turnover has become increasingly recognized. This is related to using more 1.75 mmol/L dialysate calcium concentration, more oral calcium and particularly in Africa, the unavailability of sevelamer and calcimimetic and the elevated frequency of low level of vitamin D although we live in a sunny continent.
